# HOXD-AS1 facilitates cell migration and invasion as an oncogenic lncRNA by competitively binding to miR-877-3p and upregulating FGF2 in human cervical cancer

**DOI:** 10.1186/s12885-020-07441-9

**Published:** 2020-09-25

**Authors:** Shaozheng Chen, Kejun Li

**Affiliations:** Department of Gynaecology, Tianjin Baodi People’s Hospital, No. 8 Guangchuan Road Chengguan Baodi District Tianjin, Tianjin, 301800 PR China

**Keywords:** LncRNA, HOXD-AS1, Cervical cancer, miR-877-3p, FGF2

## Abstract

**Background:**

Long non-coding RNAs (LncRNAs) are dysregulated in multiple human cancers and they are highly involved in tumor progression. Previous studies have identified the oncogenic lncRNA HOXD cluster antisense RNA 1 (HOXD-AS1) in human cancers, while its roles in cervical cancer (CC) remain unclear. Herein we intended to characterize the implication of HOXD-AS1 in CC.

**Methods:**

qRT-PCR was applied to examine the relative expression of HOXD-AS1 in CC tissues, cell lines and transfected cells. Wound healing and transwell assays were applied to detect cell migration and invasion alteration. The targeting relationship between miRNA and mRNA/lncRNA was determined by dual luciferase reporter, qRT-PCR and western blot assays.

**Results:**

HOXD-AS1 was overexpressed in CC tissues and cell lines. Its higher level predicted worse prognosis of CC patients. SiRNA mediated knockdown of HOXD-AS1 repressed CC cell migration and invasion, and its overexpression did the opposite. Mechanistically, HOXD-AS1 acted as a competing endogenous RNA (ceRNA) to sponge miR-877-3p and led to upregulation of FGF2, a target of miR-877-3p. Importantly, either miR-877-3p overexpression or FGF2 inhibition could abolish the migration and invasion promotion induced by HOXD-AS1.

**Conclusion:**

HOXD-AS1 functions as a tumor-promoting lncRNA via the miR-877-3p/FGF2 axis in CC. HOXD-AS1 might be a promising therapeutic target as well as a novel prognostic biomarker for CC.

## Background

Cervical cancer (CC) is a frequent gynecological malignancy leading the highly fatality rate in women [1]. In the worldwide, the incidence and mortality of CC have declined during the past 30 years. However, the situation remain gloomy in some developing countries with more and more young patients being observed [2–4]. Infection by human papillomavirus (HPV) is a major cause of cervical carcinogenesis [5, 6], but the detailed mechanisms stay unclear. According to the statistics, the 5-year survival rate of CC at the early stages is over 85%, whereas the survival of most CC patients at the advanced stages is unsatisfactory (lower than 40%) [7–9]. Recurrence and metastasis are the two important factors limiting the treatment effectiveness and making poor clinical outcomes of CC patients [10]. It is urgent to investigate the internal mechanisms of CC progression to develop novel anticancer strategies.

Long non-coding RNAs (LncRNAs) and microRNAs (miRNAs) are two types of non-coding RNAs (ncRNAs) both playing crucial roles various biological processes, with nucleotides longer than 200 for the former, and approximately 22 for the latter [11, 12]. Mounting evidences have proved that lncRNAs may serve as tumor promoting or suppressing factor by sponging miRNAs and then de-repressing the mRNA targets of miRNAs, forming competing endogenous RNA (ceRNA) networks in cancer cells [13–15]. Notably, many lncRNAs are known to be abnormally expressed and have high potential to be applied in cancer diagnosis, prognosis and therapy [16, 17]. Investigating the roles of tumor-related lncRNAs provide more possibilities for anticancer treatment in future.

HOXD cluster antisense RNA 1 (HOXD-AS1) is newly identified. It locates on human chromosome 2q31.2 and is transcribed from the HOXD gene cluster in an antisense manner [18]. Previous studies have demonstrated its oncogenic activities in cancers like glioma, ovarian cancer and lung cancer by affecting metastasis [19–21]. Scholars also found that HOXD-AS1 could regulate intracellular gene expression by acting as a ceRNA, in which it sponges miRNAs like miR-130a or miR-608 [19, 20]. Nevertheless, the expression status and roles of HOXD-AS1 in CC stay unclear. In the present study, we comprehensively explored its expression in paired CC tissues, analyzed its clinical significance, verified its biological functions and dig out the possible molecular mechanisms. Our findings supported the oncogenic roles of HOXD-AS1 in CC through the miR-877-5p/FGF2 axis. Our study provides new clues into the function of HOXD-AS1 and uncovers its potential as both prognostic factor and therapeutic target in for CC.

## Methods

### Patients and tissues

CC tissues and the matched non-cancerous normal tissues were received from 40 patients in the Tianjin Baodi People’s Hospital from January 2018 to December 2019. The matched non-cancerous normal tissues were obtained at a distance of 5 cm from cancerous tissues. Clinical specimens were immediately frozen in liquid nitrogen and stored at − 80 °C for further experiments. The current study was carried out in accordance with the guidelines by the Ethics and Scientific Committee of Tianjin Baodi People’s Hospital. Written informed consent was obtained from all patients involved in this study.

### Cell culture

Four CC cell lines (SiHa, CaSki, Hela, C-33A) and one normal cervical epithelial cell line (Ect1/E6E7) were purchased obtained from the Cell Bank of Institute of Biochemistry and Cell Biology, Chinese Academy of Sciences (Shanghai, China). Cells were cultured in 1640 or DMEM medium (Thermo Fisher Scientific, Waltham, MA, USA) supplemented with 10% fetal bovine serum (FBS, Thermo Fisher Scientific), 100 U/ml penicillin, and 100 μg/ml streptomycin (Thermo Fisher Scientific). They were cultured at 37 °C in a humidified incubator with 5% CO_2_.

### SiRNAs, miRNAs and transfection

HOXD-AS1 siRNAs, miR-877-3p mimics and inhibitor, negative control oligos were all ordered from GenePharma (Shanghai, China). SiRNAs, miRNA mimics or inhibitor were transfected into CC cells with Lipofectamine™ 2000 Transfection Reagent (Thermo Fisher Scientific) according to the manufacturer’s standard protocol.

### Quantitative real-time PCR

Total RNAs from CC tissues or transfected cells were extracted by using Trizol reagent (Thermo Fisher Scientific). 2 μg of RNA were reverse transcribed to detect HOXD-AS1 and GAPDH levels using the general reverse transcription kit (Promega, Madison, WI, USA). 1 μg of RNA were reverse transcribed to detect examine miR-877-3p and U6B levels using the specific primer kit (MQPS0002242–1-100 and MQPS0000002–1-100, RiboBio, Guangzhou, China). qRT-PCR was performed on the Applied Biosystems 7900HT Fast Real-Time PCR System using a standard protocol. The results were normalized to GAPDH or U6B. All the experiments were performed in three times as per the manufacturer’s instructions. The expression fold changes were calculated using the 2^-ΔΔCt^ method. The primer sequences used for qRT-PCR were as follows:

FGF2:

5′-ATGTAGAAGATGTGACGCCG-3′

5′-GGTTCACGGATGGGTGTCT-3′

HOXD-AS1:

5′-TCCTCAGGTCTAAGGACGGG-3′

5′-AGCGGAAGAGTAGGTCTGGT-3′

GAPDH:

5′-GTCAAGGCTGAGAACGGGAA-3′

5′-AAATGAGCCCCAGCCTTCTC-3′

### Wound healing assay

When the transfected cells were grown up to 90% confluence, cell monolayer was scraped in straight lines using an 1 ml pipette tip and washed with PBS twice. Then cells were further cultured in serum-free culture medium with 1% penicillin/streptomycin. Images were captured at 0 h or 48 h following the initial scratch at five random areas for each wound. ImageJ software (NIH, Bethesda, MD, USA) was used to calculate cell migration rate as: (the original wound width - the actual wound width) / (the original wound width).

### Transwell assay

Cell invasion ability was determined by transwell assay. Briefly, cells were seeded into 24-well upper chambers (Corning, NY, USA) pre-coated with Matrigel (Millipore, MA, USA) in serum-free medium. After 48 h, the invasive cells were fixed with 4% paraformaldehyde, followed by staining with 0.25% crystal violet solution (Sigma-Aldrich Co., St Louis, MO, USA) for 20 min. Then stained cells were observed and counted under an inverted microscope (Nikon, Japan), and five different microscopic views were selected randomly for analysis.

### Vector construction

The wild type sequence of HOXD-AS1 was synthesized by BGI (The Beijing Genomics Institute, Beijing, China) and cloned into the pcDNA3.1 vector (Thermo Fisher Scientific). The wild type or mutant type of 3′-UTR region of FGF2 mRNA was cloned into the pmirGLO vector (Promega). The wild type or mutant truncated sequence of HOXD-AS1 was synthesized by BGI and cloned into the pmirGLO vector.

### Dual luciferase reporter assay

Hela cells were co-transfected with miR-877-3p mimics as well as wild type or mutant HOXD-AS1. Relative luciferase activity was measured on a dual-luciferase reporter system (Promega) as per the manufacturer’s instructions. Data were calculated as the ratio of renilla to firefly luciferase activity. Luciferase reporter assays to validate the direct binding of miR-877-3p to FGF2 3′-UTR were also performed as described above.

### Western blot analysis

Total protein was extracted from cells using RIPA buffer (Beyotime Biotechnology, Nanjing, China) following the manufacturer’s instructions. Protein lysates were separated by 10% SDS-PAGE (sodium dodecyl sulfate polyacrylamide gel electrophoresis) and transferred to PVDF membranes. The membranes were then blocked with PBS containing 0.1% Triton X-100 and 5% slim milk at 25 °C for 1 h, before being incubated with anti-FGF2 or anti-β-actin antibody (Santa Cruz, Dallas, TX, USA) at 4 °C overnight. After washing, the membranes were incubated with HPR-conjuncted secondary antibodies at 25 °C for 1 h. Signal detection was carried out using an enhanced chemiluminescence detection system (Pierce, Rockford, IL, USA).

### Statistical analysis

Data was shown as mean ± standard deviation. All experiments were performed at least three times. Analyses were done with the GraphPad Prism v8 software (GraphPad Software, Inc., San Diego, CA), using the Student’s t-test for comparisons between two groups or one-way ANOVA for multiple comparisons. Survival analysis was determined by Kaplan-Meier method and log-rank test. *P* value < 0.05 was considered statistically significant.

## Results

### Increased expression of HOXD-AS1 is correlated with poor prognosis of CC patients

**Fig. 1:**
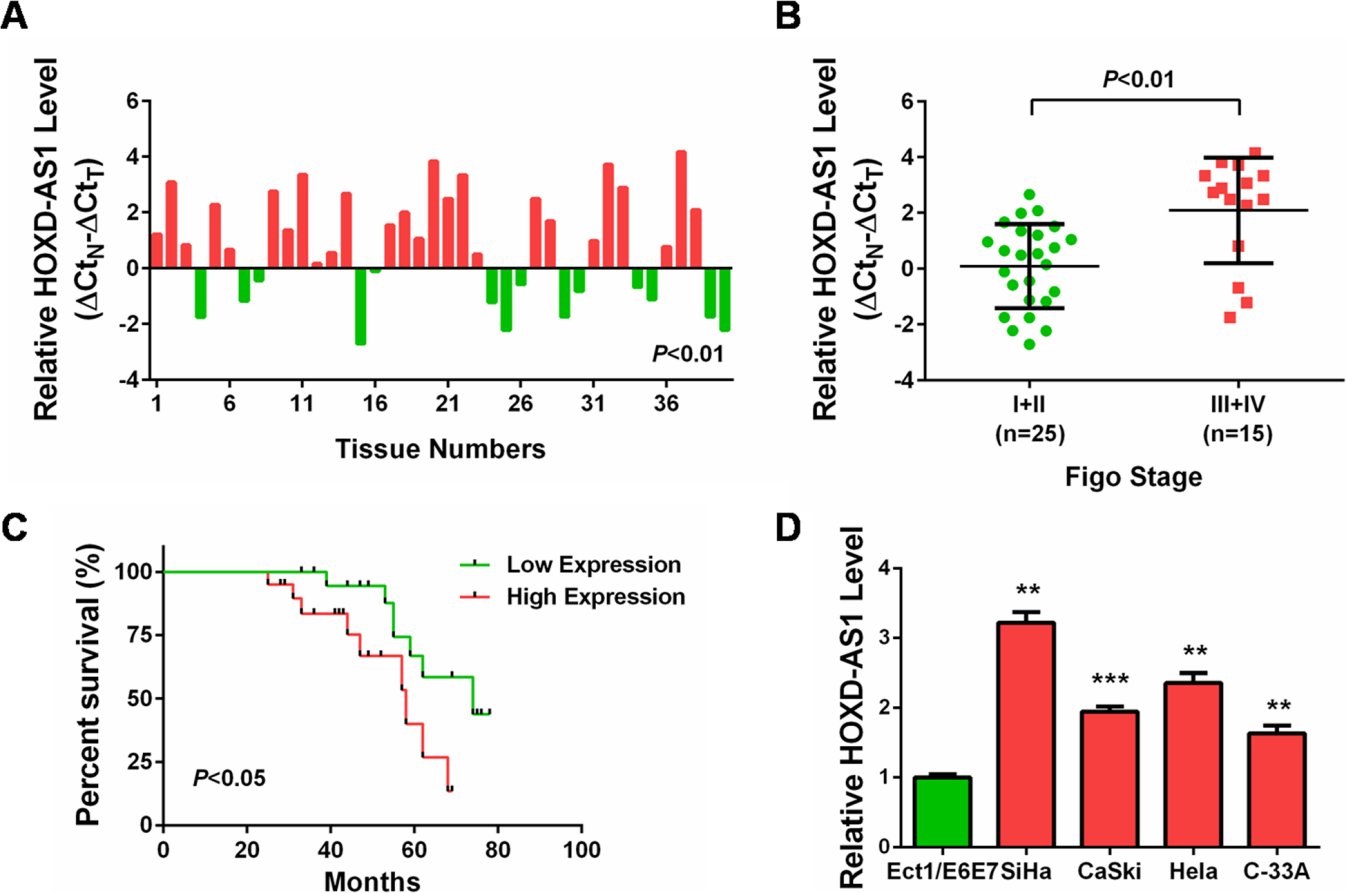
HOXD-AS1 was upregulated and correlated with worse prognosis in CC. **a** qRT-PCR analysis demonstrated that lncRNA HOXD-AS1 was significantly upregulated in cancerous tissues compared with in matched normal tissues. Expression levels of HOXD-AS1 were normalized to that of GAPDH. Data were calculated from triplicates. Bar value is the difference of HOXD-AS1 expression levels between tumors (T) and matched normal tissues (N) from the same patient. Bar value ≤ − 1 represents HOXD-AS1 is decreased in tumors. Bar value ≥1 represents that HOXD-AS1 is increased in tumors. **b** qRT-PCR analysis demonstrated that lncRNA HOXD-AS1 expression was higher in high stage (III + IV) CC tissues compared with in low stage (I + II) CC tissues. **c** Kaplan-Meier method with log-rank test indicated that patients with higher HOXD-AS1 expression had worse overall survival than those with lower HOXD-AS1 expression. **d** qRT-PCR analysis demonstrated that lncRNA HOXD-AS1 was significantly upregulated in CC cells (SiHa, CaSki, Hela, C-33A) compared with normal cervical epithelial cells (Ect1/E6E7). **: *P* < 0.01, ***: *P* < 0.001

We firstly detected the expression of HOXD-AS1 in 40 pairs of CC tissues (cancerous and the matched normal tissues) by qRT-PCR. The results shown in Fig. 1a revealed that among all the 40 pairs of CC tissues, 26 pairs (65.0%) expressed positive HOXD-AS1 expression (ΔΔCt> 0), and 19 pairs (47.5%) showed upregulation of HOXD-AS1 (ΔΔCt≥1) in CC tissues compared with the normal tissues. According to the classification of the clinical stages of these CC patients, we found that HOXD-AS1 expression was higher in advanced CC stages (III + IV vs. I + II; Fig. 1b). To further analyze the prognostic significance of HOXD-AS1 in CC, we divided these CC tissues into two subgroups on the basis of the median expression value, and Kaplan-Meier survival analysis was conducted. As shown in Fig. 1c, patients with higher HOXD-AS1 level presented worse overall survival than those with lower HOXD-AS1 level. Furthermore, the expression exploration in CC cell lines also showed that HOXD-AS1 expression was increased in CC cell lines compared with the normal cervical epithelial cell line (Ect1/E6E7; Fig. 1d). Therefore, these results strongly suggested that HOXD-AS1 might be implicated in CC carcinogenesis, and its higher level predicted the worse outcome of the patients.

### HOXD-AS1 promotes CC cell migration and invasion in vitro

**Fig. 2:**
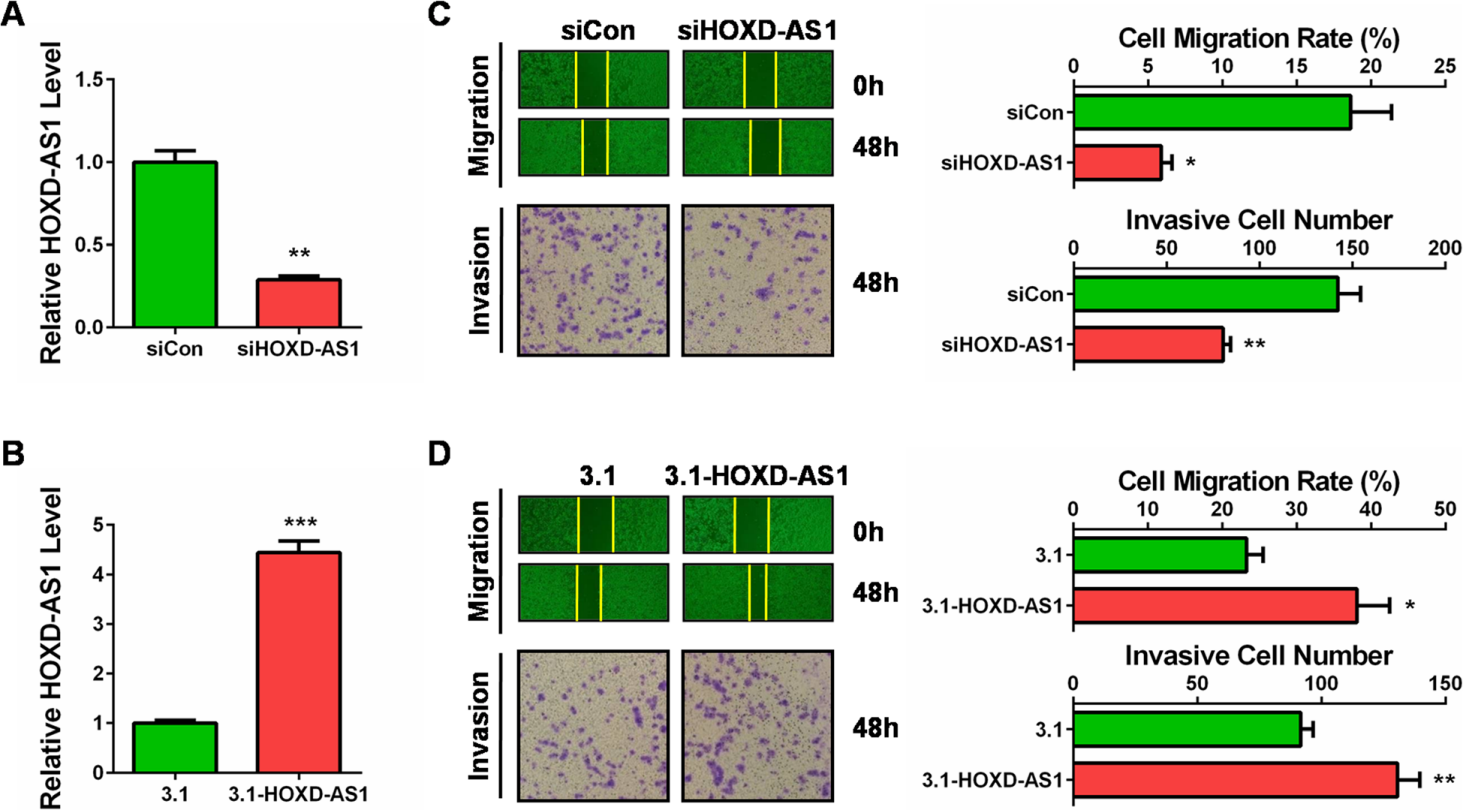
HOXD-AS1 promoted cell migration and invasion in CC cells. **a** The transfection efficiency of siHOXD-AS1 was confirmed by qRT-PCR. **b** The transfection efficiency of 3.1-HOXD-AS1 was confirmed by qRT-PCR. **c** Hela cells transfected with siHOXD-AS1 showed reduced migration and invasion abilities than control cells measured by wound healing and transwell assays, respectively. The right panel showed the quantitative results. **d** Hela cells transfected with 3.1-HOXD-AS1 showed enhanced migration and invasion abilities than control cells measured by wound healing and transwell assays, respectively. The right panel showed the quantitative results. *: *P* < 0.05, **: *P* < 0.01, ***: *P* < 0.001

To explore whether HOXD-AS1 exerted some effects on the mobility of CC cells, we evaluated the migration and invasion capabilities of Hela cells after transfection with its siRNAs (knockdown) or coding plasmid (overexpression). As shown in Fig. 2a and b, siRNAs specifically targeting HOXD-AS1 efficiently silenced its endogenous expression, while the coding plasmid effectively overexpressed its level in Hela cells. By transwell migration and invasion assays, we found that knockdown of HOXD-AS1 significantly repressed cell migration and invasion of Hela cells, respectively (Fig. 2c). To the opposite, forced expression of HOXD-AS1 facilitated cell migration and invasion of Hela cells (Fig. 2d). These data indicated that HOXD-AS1 itself was a metastasis promoter by accelerating migration and invasion of CC cells.

### HOXD-AS1 induces FGF2 expression by competitively sponging miR-877-3p in Hela cells

**Fig. 3:**
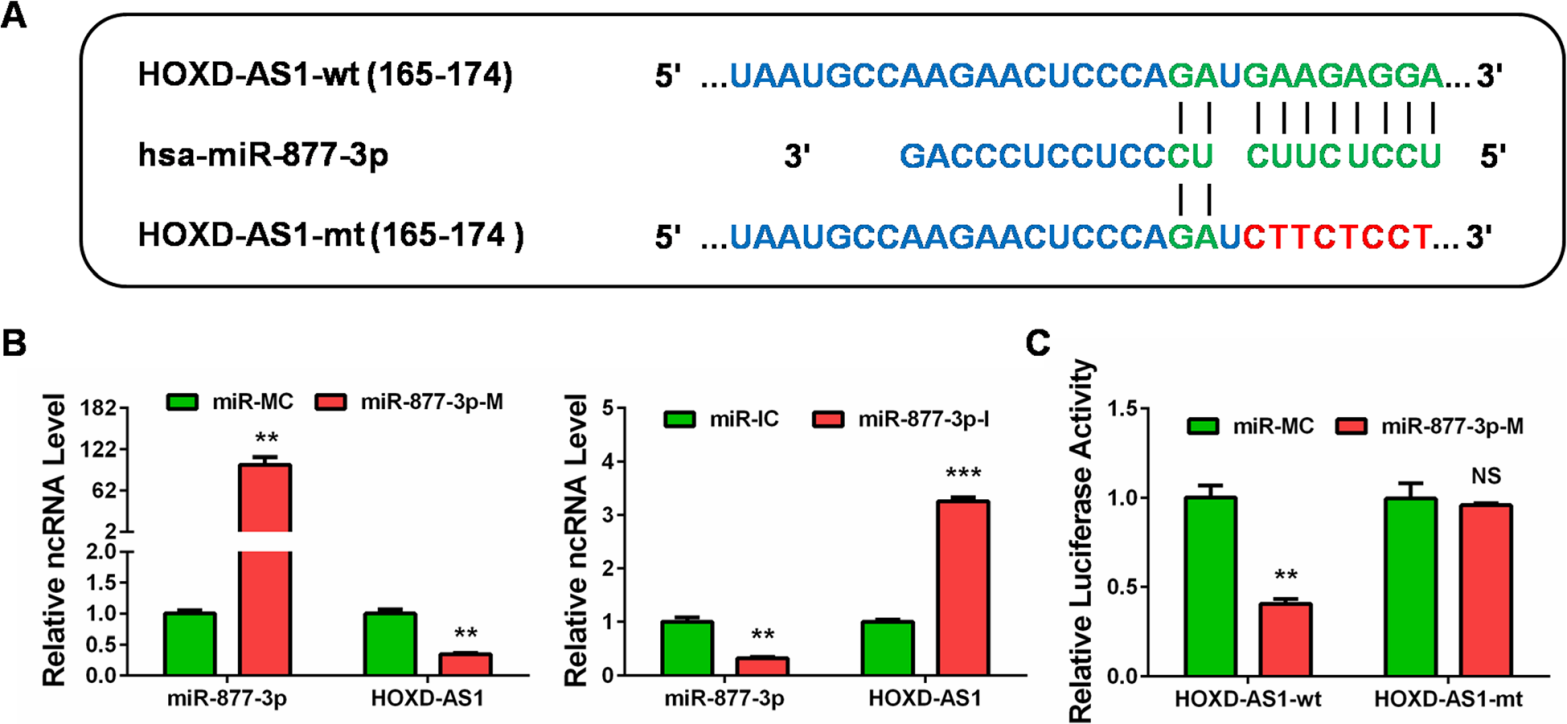
HOXD-AS1 directly interacted with miR-877-3p in CC cells. **a** The wild type (in green) and mutant type (in red) binding sites between HOXD-AS1 and miR-877-3p were obtained from the bioinformatics software LncBase v.2. **b** qRT-PCR was performed to measure the effect of miR-877-3p on HOXD-AS1 expression in Hela cells transfected with miR-877-3p mimics (miR-877-3p-M) and miR-877-3p inhibitor (miR-877-3p-M-I). **c** Dual luciferase reporter activity was detected after co-transfection with miR-877-3p and wild type HOXD-AS1 (HOXD-AS1-wt) or mutant type HOXD-AS1 (HOXD-AS1-mt). **: *P* < 0.01, ***: *P* < 0.001

It is widely recognized that some lncRNAs serve as ceRNAs for miRNAs thus to control gene expression [13]. To screen the putative miRNAs interacting with HOXD-AS1, we utilized the online bioinformatics tool (DIANA tools, LncBase Predicted v.2, http://carolina.imis.athena-innovation.gr/diana_tools/web/index.php) and found that miR-877-3p might bind to HOXD-AS1 (Fig. 3a). qRT-PCR showed that miR-877-3p mimics repressed the expression of HOXD-AS1, while miR-877-3p inhibitor markedly increased HOXD-AS1 expression in Hela cells (Fig. 3b). Then we constructed luciferase reporters containing the wild type miR-877-3p binding site (HOXD-AS1-wt) or the mutant type one (HOXD-AS1-mt). Co-transfection of miR-877-3p mimics and HOXD-AS1-wt largely inhibited the luciferase activity compared with the control group. However, miR-877-3p mimics failed to inhibit the luciferase activity of the HOXD-AS1-mt group (Fig. 3c). These results demonstrated that HOXD-AS1 interacted with miR-877-3p in CC cells.

**Fig. 4:**
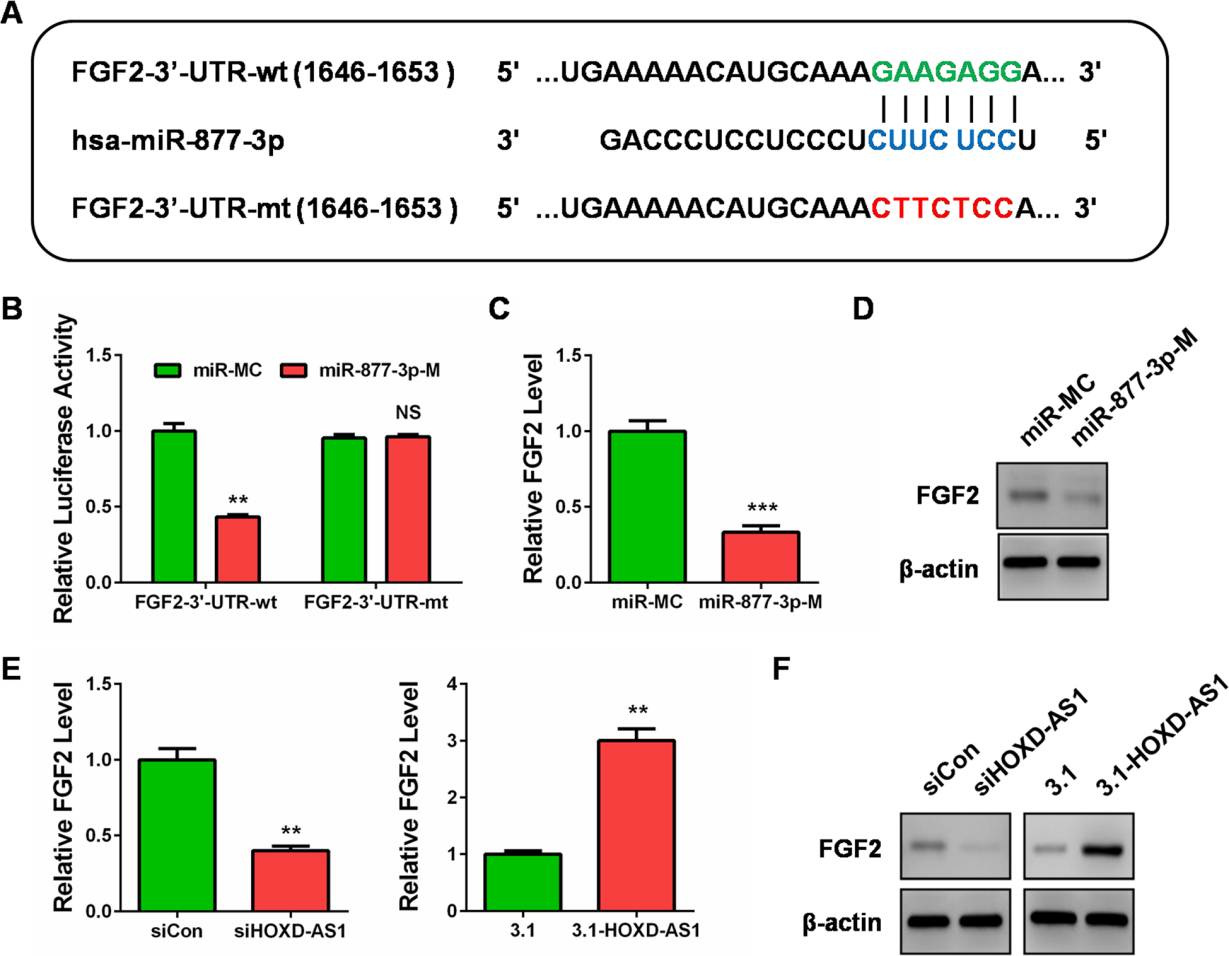
HOXD-AS1 induced FGF2 expression by competitively sponging miR-877-3p in CC cells. **a** The wild type (in green) and mutant (in red) binding sites between FGF2–3′-UTR and miR-877-3p were obtained from the bioinformatics software TargetScan Human Release 7.2. **b** Luciferase activity was measured in Hela cells co-transfected with wild type FGF2 3’UTR (FGF2–3′-UTR-wt) or mutant type FGF2 3’UTR (FGF2–3′-UTR-wt) and miR-877-3p using the dual luciferase reporter assay. **c** qRT-PCR analysis of FGF2 mRNA level following treatment of Hela cells and with miR-877-3p mimics (miR-877-3p-M). **d** Western blot analysis of FGF2 protein level following treatment of Hela cells with miR-877-3p mimics (miR-877-3p-M). **e** qRT-PCR analysis of FGF2 mRNA level in Hela cells with HOXD-AS1 knockdown or overexpression (si HOXD-AS1 or 3.1-HOXD-AS1). **f** Western blot analysis of FGF2 protein level in Hela cells with HOXD-AS1 knockdown or overexpression (si HOXD-AS1 or 3.1-HOXD-AS1). **: *P* < 0.01, ***: *P* < 0.001, NS: not significant

To further understand the mechanism of HOXD-AS1 in CC cells, we predicted the mRNA targets of miR-877-3p by using the online Targetscan tool (http://www.targetscan.org/vert_70/) and found that fibroblast growth factor 2 (FGF2), a member of the fibroblast growth factor (FGF) family, might be a target of miR-877-3p (Fig. 4a). The similar luciferase reporter assay was performed, and the results showed that miR-877-3p mimics inhibited the wild type FGF2–3′-UTR (pmirGLO-FGF2–3′-UTR-wt) activity, whereas it failed to inhibit luciferase activity of the mutant type FGF2–3′-UTR (pmirGLO- FGF2–3′-UTR-mt) in which the binding site of miR-877-3p was mutated (Fig. 4a and b). qRT-PCR and western blot confirmed the targeting of FGF2 by miR-877-3p, as miR-877-3p mimics transfection led to repression of FGF2 expression in Hela cells (Fig. 4c and d). Importantly, the results also revealed that knockdown of HOXD-AS1 inhibited FGF2 expression, and HOXD-AS1 overexpression caused FGF2 upregulation at both mRNA and protein levels (Fig. 4e and f). Collectively, these results clearly implied that HOXD-AS1 could induce FGF2 expression by competitively sponging miR-877-3p in CC cells. There exists a HOXD-AS1/miR-877-3p/FGF2 ceRNA axis in CC cells.

### MiR-877-3p/FGF2 mediates the effect of HOXD-AS1 on CC cell migration and invasion

**Fig. 5:**
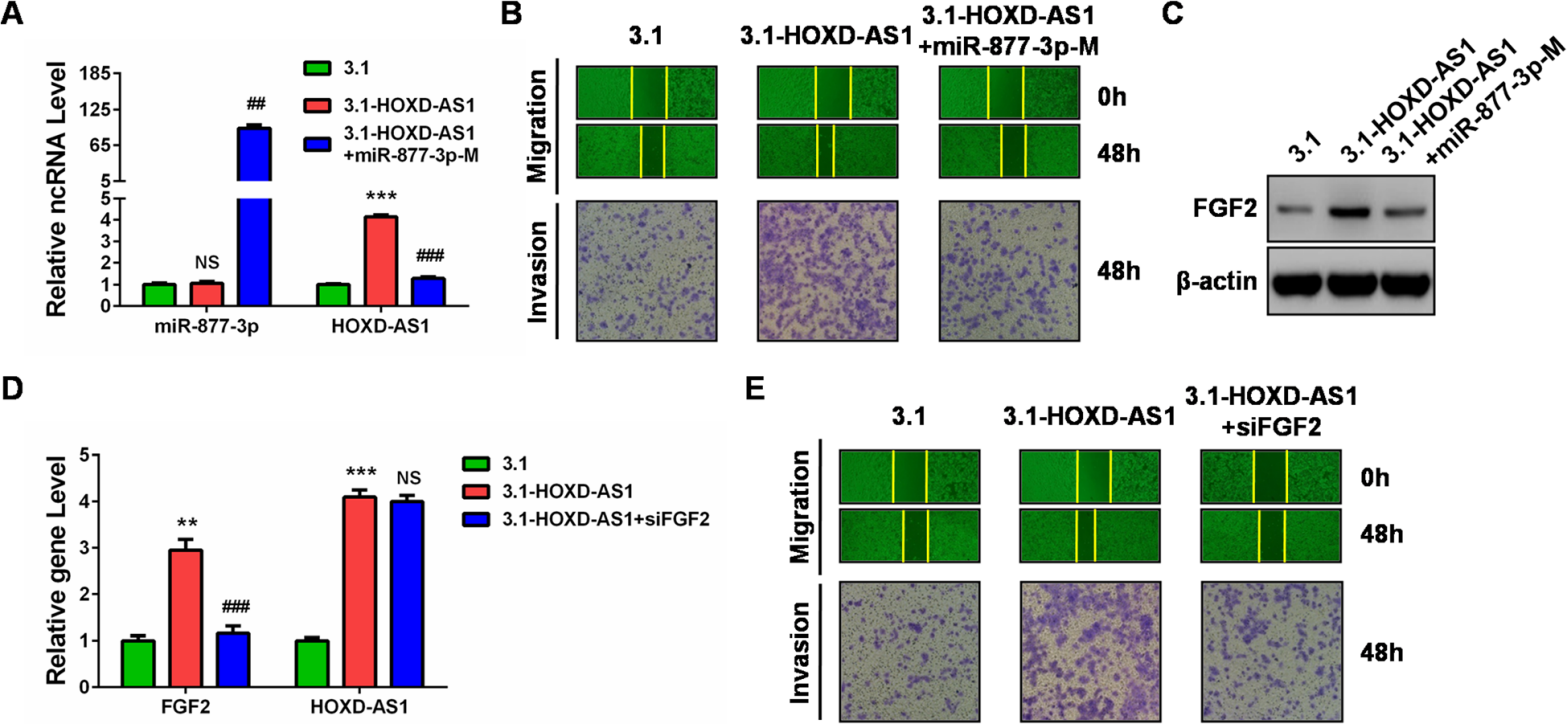
MiR-877-3p/FGF2 reversed the effects of HOXD-AS1 on CC cell migration and invasion. **a** miR-877-3p and HOXD-AS1 expressions were examined using qRT-PCR after rescuing the expression of miR-877-3p. **b** Cell migration and invasion abilities were reduced after rescuing the expression of miR-877-3p in HOXD-AS1-overexpression group by wound healing and transwell assays. **c** FGF2 protein expression was examined using western bolt analysis after rescuing the expression of miR-877-3p. **(D)** FGF2 and HOXD-AS1 expressions were examined using qRT-PCR after knocking down the expression of FGF2. **(E)** Cell migration and invasion abilities were reduced after knocking down the expression of FGF2 in HOXD-AS1-overexpression group by wound healing and transwell assays. **: *P* < 0.01, ***: *P* < 0.001, ##: *P* < 0.01 vs 3.1-HOXD-AS1 group, ###: *P* < 0.001 vs 3.1-HOXD-AS1 group, NS: not significant

As we had verified that HOXD-AS1 was a metastasis promoter by accelerating migration and invasion of CC cells, we then sought to determine whether this tumor promoting activity of HOXD-AS1 was mediated by the miR-877-3p/FGF2 axis. We restored the expression of miR-877-3p in HOXD-AS1-overexpressed Hela cells, and found that miR-877-3p restoration could reverse the promotion of HOXD-AS1 on CC cell migration and invasion (Fig. 5a and b). We also noticed that miR-877-3p restoration obviously downregulated FGF2 expression in HOXD-AS1-overexpressed Hela cells (Fig. 5c). To the other end, siRNA targeting FGF2 in HOXD-AS1-overexpressed Hela cells also recovered the migration and invasion capacities (Fig. 5d and e). Therefore, our findings suggested that the tumor promoting effects of HOXD-AS1 on CC cell migration and invasion were mediated by the miR-877-3p/FGF2 axis.

## Discussion

**Fig. 6:**
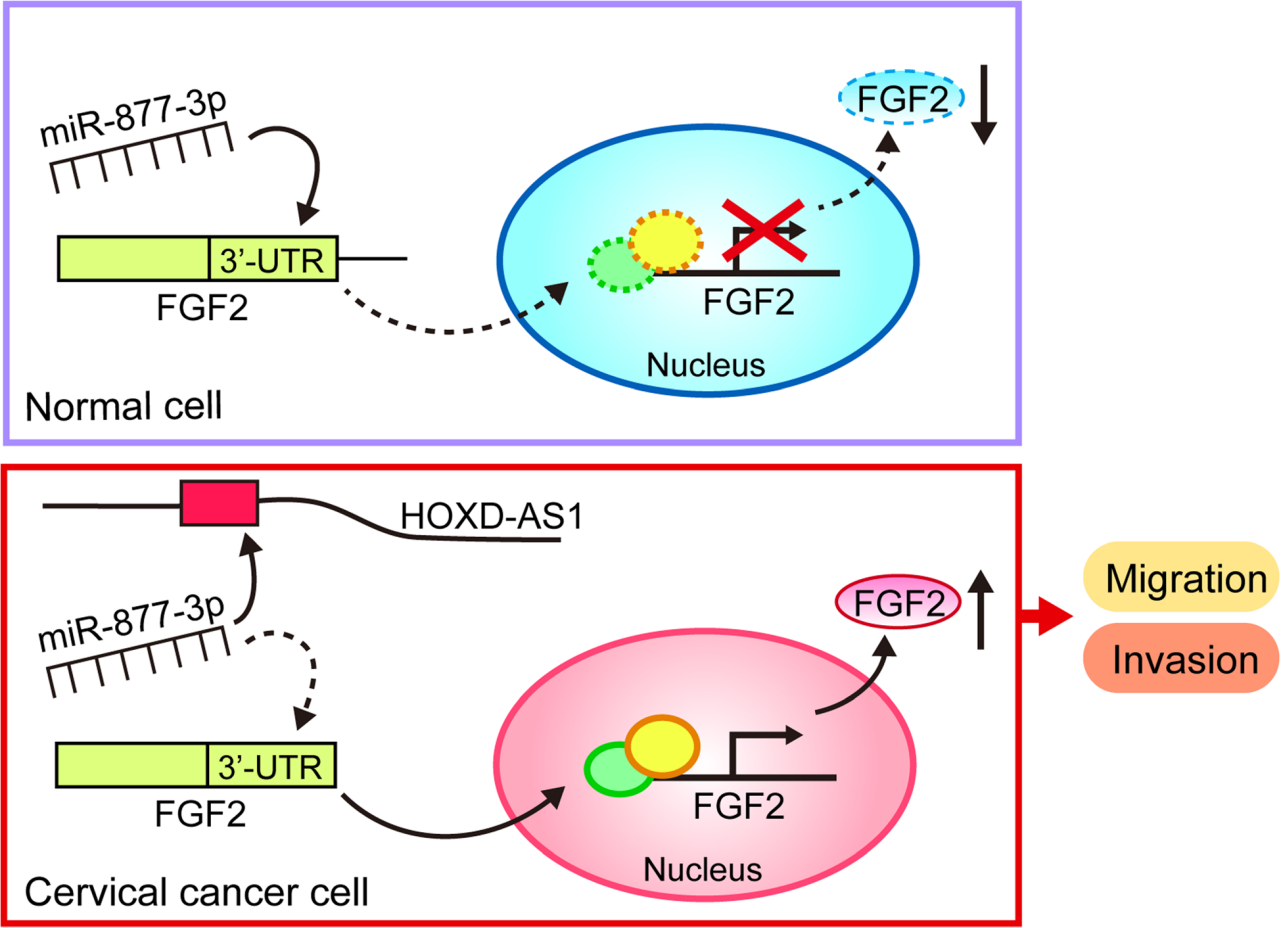
Schematic illustration of the molecular mechanism in which HOXD-AS1/miR-877-3p/FGF2 forms the ceRNA relationship in CC cells

Through a series of experiments, our study clearly demonstrated that the lncRNA HOXD-AS1 is significantly higher expressed in CC and its higher levels correlated with poor prognosis of the patients. Cellular functional and mechanistic assays verified that HOXD-AS1 serves as an oncogene by competitively binding to miR-877-3p and upregulating FGF2 to facilitate CC cell migration and invasion (Fig. 6).

Till now, a variety of lncRNAs are implicated with oncogenesis and have high potential to be used as cancer biomarkers [16, 17]. Aberrant expression of some lncRNAs is also frequently observed and they may contribute to the pathophysiology of CC [22]. In this study, we found that HOXD-AS1 was markedly overexpressed in CC tissues compared with normal tissues, extremely overexpressed in those CC tissues in advanced CC stages. Its expression status in CC cell lines also showed upregulation. Moreover, its higher level predicted the worse outcome of the patients. Based on these initial findings, we hypothesized that HOXD-AS1 was involved in CC carcinogenesis. Subsequent in vitro loss and gain-of-function studies pointed out that HOXD-AS1 promoted CC cell migration and invasion.

CeRNA is an important concept in which lncRNAs interact with miRNA thus to de-repress the mRNA targets of miRNAs [13–15]. There are numerous reports characterizing the modulation of lncRNAs as ceRNAs in CC [23–25]. Herein, the bioinformatics prediction suggested that miR-877-3p might bind to HOXD-AS1. We then proved that HOXD-AS1 might interact with miR-877-3p by qRT-PCR and luciferase reporter assays. Similarly, we also validated FGF2 as a direct target of miR-877-3p. In a word, miR-877-3p could simultaneously bind to HOXD-AS1 and FGF2. Through expression detection, we found that HOXD-AS1 could induce FGF2 expression. Therefore, there exists a HOXD-AS1/miR-877-3p/FGF2 ceRNA axis in CC cells.

As a member of the fibroblast growth factor (FGF) family, FGF2 is a key mitogen in tissue homeostasis and cancer. For instance, it impacts on cell differentiation, angiogenesis and inflammatory responses, thus contributing to tumorigenesis [26]. Notably, FGF2 facilitates cell migration and invasion in some cancers like breast, pancreatic and cervical cancers [27–29]. In this study, we confirmed the pro-metastasis function of FGF2 in CC. We further investigated the linkage of the pro-metastasis role of HOXD-AS1 with the miR-877-3p/FGF2 axis in CC cells. The function experiments showed that either miR-877-3p restoration or FGF2 knockdown could effectively reverse the promotion of HOXD-AS1 on CC cell migration and invasion. These results illustrated that the tumor promoting activities of HOXD-AS1 on CC cell migration and invasion were mediated by the miR-877-3p/FGF2 axis.

## Conclusions

Our findings revealed the oncogenic roles of HOXD-AS1 in CC cell migration and invasion, and identified the possible molecular mechanism. In future, more comprehensive studies detailing the downstream signaling and in vivo studies are both needed to warrant its roles in CC. Our findings enrich the knowledge about the function of HOXD-AS1 in CC and consider that it might be used as a promising prognostic factor and therapeutic target for CC.

## Data Availability

All data generated or analyzed during this study are included in this published article which can be further obtained from the corresponding author.

## Abbreviations

LncRNAs: Long non-coding RNAs
HOXD-AS1: HOXD cluster antisense RNA 1
CC: Cervical cancer
ceRNA: Competing endogenous RNA
HPV: Human papillomavirus
FGF: Fibroblast growth factor

## References

[1] Liontos M, Kyriazoglou A, Dimitriadis I, Dimopoulos MA, Bamias A (2019). Systemic therapy in cervical cancer: 30 years in review. Crit Rev Oncol Hematol.

[2] Vu M, Yu J, Awolude OA, Chuang L (2018). Cervical cancer worldwide. Curr Probl Cancer.

[3] Small W, Bacon MA, Bajaj A, Chuang LT, Fisher BJ, Harkenrider MM (2017). Cervical cancer: a global health crisis. Cancer..

[4] Wang L, Zhao Y, Wang Y, Wu X (2018). The role of Galectins in cervical cancer biology and progression. Biomed Res Int.

[5] Kessler TA (2017). Cervical cancer: prevention and early detection. Semin Oncol Nurs.

[6] Castellsagué X (2008). Natural history and epidemiology of HPV infection and cervical cancer. Gynecol Oncol.

[7] Franco EL, Duarte-Franco E, Ferenczy A (2001). Cervical cancer: epidemiology, prevention and the role of human papillomavirus infection. CMAJ..

[8] Bosch FX, Lorincz A, Muñoz N, Meijer CJ, Shah KV (2002). The causal relation between human papillomavirus and cervical cancer. J Clin Pathol.

[9] Jensen PT, Schnack TH, Frøding LP, Bjørn SF, Lajer H, Markauskas A (2020). Survival after a nationwide adoption of robotic minimally invasive surgery for early-stage cervical cancer - a population-based study. Eur J Cancer.

[10] Li H, Wu X, Cheng X (2016). Advances in diagnosis and treatment of metastatic cervical cancer. J Gynecol Oncol.

[11] Zhang X, Hong R, Chen W, Xu M, Wang L (2019). The role of long noncoding RNA in major human disease. Bioorg Chem.

[12] Vishnoi A, Rani S (2017). MiRNA biogenesis and regulation of diseases: an overview. Methods Mol Biol.

[13] Lou W, Ding B, Fu P (2020). Pseudogene-derived lncRNAs and their miRNA sponging mechanism in human cancer. Front Cell Dev Biol.

[14] Ye Y, Shen A, Liu A (2019). Long non-coding RNA H19 and cancer: a competing endogenous RNA. Bull Cancer.

[15] Abdollahzadeh R, Daraei A, Mansoori Y, Sepahvand M, Amoli MM, Tavakkoly-Bazzaz J (2019). Competing endogenous RNA (ceRNA) cross talk and language in ceRNA regulatory networks: a new look at hallmarks of breast cancer. J Cell Physiol.

[16] Wang L, Cho KB, Li Y, Tao G, Xie Z, Guo B (2019). Long noncoding RNA (lncRNA)-mediated competing endogenous RNA networks provide novel potential biomarkers and therapeutic targets for colorectal cancer. Int J Mol Sci.

[17] Sarfi M, Abbastabar M, Khalili E (2019). Long noncoding RNAs biomarker-based cancer assessment. J Cell Physiol.

[18] Li L, Wang Y, Zhang X, Huang Q, Diao Y, Yin H (2018). Long non-coding RNA HOXD-AS1 in cancer. Clin Chim Acta.

[19] Chen Y, Zhao F, Cui D, Jiang R, Chen J, Huang Q (2018). HOXD-AS1/miR-130a sponge regulates glioma development by targeting E2F8. Int J Cancer.

[20] Wang Y, Zhang W, Wang Y, Wang S (2018). HOXD-AS1 promotes cell proliferation, migration and invasion through miR-608/FZD4 axis in ovarian cancer. Am J Cancer Res.

[21] Xia H, Jing H, Li Y, Lv X (2018). Long noncoding RNA HOXD-AS1 promotes non-small cell lung cancer migration and invasion through regulating miR-133b/MMP9 axis. Biomed Pharmacother.

[22] Aalijahan H, Ghorbian S (2019). Long non-coding RNAs and cervical cancer. Exp Mol Pathol.

[23] Luan X, Wang Y (2018). LncRNA XLOC_006390 facilitates cervical cancer tumorigenesis and metastasis as a ceRNA against miR-331-3p and miR-338-3p. J Gynecol Oncol.

[24] Rui X, Xu Y, Jiang X, Ye W, Huang Y, Jiang J (2018). Long non-coding RNA C5orf66-AS1 promotes cell proliferation in cervical cancer by targeting miR-637/RING1 axis. Cell Death Dis.

[25] Peng L, Yuan X, Jiang B, Tang Z, Li GC (2016). LncRNAs: key players and novel insights into cervical cancer. Tumour Biol.

[26] Coleman SJ, Chioni AM, Ghallab M, Anderson RK, Lemoine NR, Kocher HM (2014). Nuclear translocation of FGFR1 and FGF2 in pancreatic stellate cells facilitates pancreatic cancer cell invasion. EMBO Mol Med.

[27] Turner N, Grose R (2010). Fibroblast growth factor signalling: from development to cancer. Nat Rev Cancer.

[28] Chen X, Zhao H, Chen C, Li J, He J, Fu X (2020). The HPA/SDC1 axis promotes invasion and metastasis of pancreatic cancer cells by activating EMT via FGF2 upregulation. Oncol Lett.

[29] Duan H, Li X, Chen Y, Wang Y, Li Z (2019). LncRNA RHPN1-AS1 promoted cell proliferation, invasion and migration in cervical cancer via the modulation of miR-299-3p/FGF2 axis. Life Sci.

